# Generation and properties of the new asphalt binder model using molecular dynamics (MD)

**DOI:** 10.1038/s41598-021-89339-5

**Published:** 2021-05-10

**Authors:** Hui Yao, Junfu Liu, Mei Xu, Andreas Bick, Qing Xu, Jinxi Zhang

**Affiliations:** 1 Beijing Key Laboratory of Traffic Engineering, Faculty of Architecture, Civil and Transportation Engineering, Beijing University of Technology, No.100, Pingleyuan, Chaoyang, Beijing, 100124 China; 2 Scienomics SARL, 16 rue de l’Arcade, 75008 Paris, France

**Keywords:** Civil engineering, Mechanical properties

## Abstract

Asphalt binder is the main material for road pavement and building construction. It is a complex mixture composed of a large number of hydrocarbons with different molecular weights. The study of asphalt binders and asphalt concretes from a molecular perspective is an important means to understand the intricate properties of asphalt. Molecular dynamics simulation is based on Newton’s law and predicts the microscopic performance of materials by calculating the intra- and intermolecular interactions. The asphalt binder can be divided into four components: saturates, aromatics, resins, and asphaltenes (SARA). A new molecular model of asphalt was proposed and verified in this study. Eight molecules selected from the literature were used to represent the four components of asphalt. The AMBER Cornell Extension Force Field was applied in this study to model building and the calculation of properties. The density of the asphalt model was calculated and compared with experimental results for validity verifications. The results show that the purposed model can be used to calculate the microscopic properties of the asphalt binder because the density of the model is close to the real value in the field. Besides, the proportions of different molecules in the model were adjusted to predict the relationship between the asphalt binder density and the hydrocarbon ratios and heteroatom contents of the molecular model. Moreover, the glass transition temperature of the asphalt binder model is predicted by the simulation of the heating process. The range of the glass transition temperature is determined by calculating the relationship between specific volume and temperature, and the calculated range is close to the experimental value.

## Introduction

As a complex product of crude distillation, asphalt is extensively used in road pavement, building construction, and industry application^1^. Therefore, exploring the performance, properties, and composition of asphalt materials is an important topic. Various compounds in asphalt are mainly composed of carbon, hydrogen, and some other trace elements such as oxygen, nitrogen, sulfur, and metallic elements. Heteroatoms affect the properties of asphalt binder, but the content is minimal, with oxygen, nitrogen, and sulfur atoms in the total range of 1–6% and metal atoms less than 1%. Hydrocarbons formed from carbon and hydrogen make up most of the asphalt molecules, and their content is as high as 90 to 95 percent^2–4^. Although there are a huge number of organic molecules in asphalt, it can be classified into several different types of compounds according to molecular weight, functional group, and polarity^5^. The compositional analysis of asphalt plays an important role in the production, transportation, processing, and engineering application of asphalt binder. The main method to distinguish the components in petroleum is to separate them into saturates hydrocarbons, aromatics hydrocarbons, resins, and asphaltenes. The asphaltene with large volume, complex structure, and high polarity contributes greatly to the viscosity of asphalt while maltenes are less polar and smaller in size. This separation method is the basis for higher-purity compositions, such as gas chromatography (GC) and gas chromatography-mass spectrometry (GS-MS)^6^. GC and GS-MS are usually applied for the extraction of different functional groups and molecules in the asphalt component to obtain the detailed molecular composition of the asphalt binder. In the determination of asphalt components, the Corbett method is highly recognized, which separates asphalt into four parts, such as asphaltene, saturates components, naphthene aromatics, and polar aromatic^7^. The Corbett method consists of two-part: firstly, recovery asphaltene based upon solvent precipitation and extract saturates, naphthene-aromatics, and polar-aromatics by elution-adsorption chromatography. Then, the average chemical structures of these compositions were defined by the densiometric method^8^.

Although the molecular composition of asphalt is extremely complex, many scholars are still committed to this research to deeply understand the performance of asphalt from a microscopic perspective. In the early research, Jennings et al. selected eight asphalt binders as a standard sample in the Strategic Highway Research Program (SHRP). The Nuclear Magnetic Resonance (NMR) spectroscopic was used to analyze the molecular parameters such as percentage of aromatic carbon, elementary composition. Besides, the average molecular structures were proposed in the research^9^. Asphaltenes are polyarene compounds consisting of aromatic cores and aliphatic side chains, and usually containing heteroatoms such as sulfur, nitrogen, and oxygen^10^. Artok et al. discussed the structure of asphaltene molecular based on the data of NMR and built several models of asphaltene molecular^11^. Murgich et al. have built several average molecular models of resins and asphaltenes, then explored the aggregation of asphaltenes^12^. Groenzin and Mullins recommended several molecular models of asphaltenes, then, used fluorescence depolarization measurements to compare the model compounds and determine dimensions^13^. Then, Mullins proposed a modified Yen model that has been proven to be successful in studying interfacial phenomena of asphaltene^14^. Based on the molecular model of asphalt components, more researchers used the molecular simulation method to verify the effectiveness of the asphalt model and put forward a more realistic asphalt model by comparing the calculation result and experimental data. Besides, the deep learning method was applied to study organic structure molecules and verify them with experimental data^15,16^. Researchers also studied the changes of the asphalt molecular model and the aging process by the artificial neural network method. Therefore, it is important means of studying asphalt material to carry out computation simulation research based on experimental data^17^.

Molecular dynamics (MD) simulation is a computer simulation method based on the principle of statistic mechanics and thermomechanical theory, which can simulate the interactions and behaviors of all-atom and molecules in a given period. After the MD simulation, the chemo-physical and thermodynamic properties of the molecular model can be calculated. The important purpose of MD simulation is to give a mechanics explanation of material behaviors in nanoscales^18^. Besides, this method has been proved to be an effective method to study the properties of asphalt binder, such as oxidative age, moisture sensitivity, and physical characteristics^19,20^. With the development of computing science and computer technology, many programs, and software for molecular simulation have been created. The most representative is the Large-scale Atomic/Molecular Massively Parallel Simulator (LAMMPS) for MD simulation and Towhee for Monte Carlo simulation.

Many researchers studied the molecular composition and MD simulation of asphalt. Zhang and Greenfield purposed a three components model of asphalt and then calculated the properties of the model by molecular simulation. Two different asphaltene molecular models were selected from the literature, both of which had medium-sized aromatic cores. The aliphatic side chain of asphaltene1 is longer than asphaltene2. In addition, the saturate and the aromatic compositions of this model are represented by n-docosane (n-C_22_H_46_) and 1,7-dimethylnaphthalene, respectively^7^. The calculated molecular number and mass fraction are matched with the reference experimental data. And later, the physical, mechanical, rheological, and microstructure of the model asphalt system are studied by molecular simulation^21,22^. Recently, a new molecular model was purposed by Li and Greenfield to characterize the properties of the asphalt binder. The asphalt model contains four groups: asphaltenes, saturates, polar aromatics, and naphthene aromatics, and 12 components were chosen in this model. The asphaltene in this model was obtained from the modified Mullins model. The side chains of asphaltene in the Mullins model were repositioned to reduce the higher internal energy of the molecule due to the pentane effect. In this study, the OPLS-aa (all-atom optimized parameters for liquid simulations) Force Field was utilized to calculate and analyze the properties of model asphalt. The results show that the parameters of the OPLS-aa force field can accurately calculate and predict the molecular properties of asphalt. Besides, the molecular model also overcomes the defects of the previous model in terms of density prediction and relaxation time^23^. The model proposed by Li et al. was successful applied to many nanoscales asphalt research, which also indicates that an effective molecular model of asphalt binder is important to the performance research of asphalt at the molecular scale^24–26^.

The accuracy of the molecular model is determined by the parameter describing the atomic interaction that is also named the force field parameter. Simulation under the appropriate force field can not only conform to the experimental result but also predict the properties and mechanical behavior of materials more accurately^27^. Various kinds of force fields were applied in the molecular simulation of the asphalt model and the calculation of various properties of asphalt, such as AMBER Cornell Extension, OPLS-AA, DREIDING, COMPASS, and ReaxFF^3,7,28–30^. However, a few types of research compared the mechanical behavior of asphalt molecules under different force fields. In this study, DREIDING and AMBER Cornell Extension Force Field were selected to verify the density of the proposed asphalt model, and then, the suitable force field was selected for subsequent research.

The main objective of this study is to integrate the various molecular models mentioned in the literature to produce a new asphalt model, and to verify the proposed asphalt molecular model with experimental data. Eight kinds of molecules in the literature were selected, and the proportion of molecules selected was adjusted to make them more consistent with the actual situation. The DREINDING Force Field and Amber Cornell Extension Force Field were applied to the model asphalt to calculate the density of the asphalt model and confirm the validity of the purposed model. Then, the density values of the asphalt models were compared to the testing result and a more suitable force field is selected to predict the glass transition temperature of the asphalt binder model.

## Molecular model and simulation method

### Molecular selection

The molecular composition of the asphalt binder is complex. The components of the asphalt binder are distinguished by polarity, acidity or alkalinity and molecular size, and so on, in order to characterize the molecular structure of asphalt. Therefore, asphalt binders are usually separated into four fractions, such as saturates, asphaltene, resin, and aromatics. Different components have different physical properties, solubility, and polarity. In this study, eight kinds of molecular were chosen to represent the four fractions, respectively.

### Asphaltene

**Figure 1:**
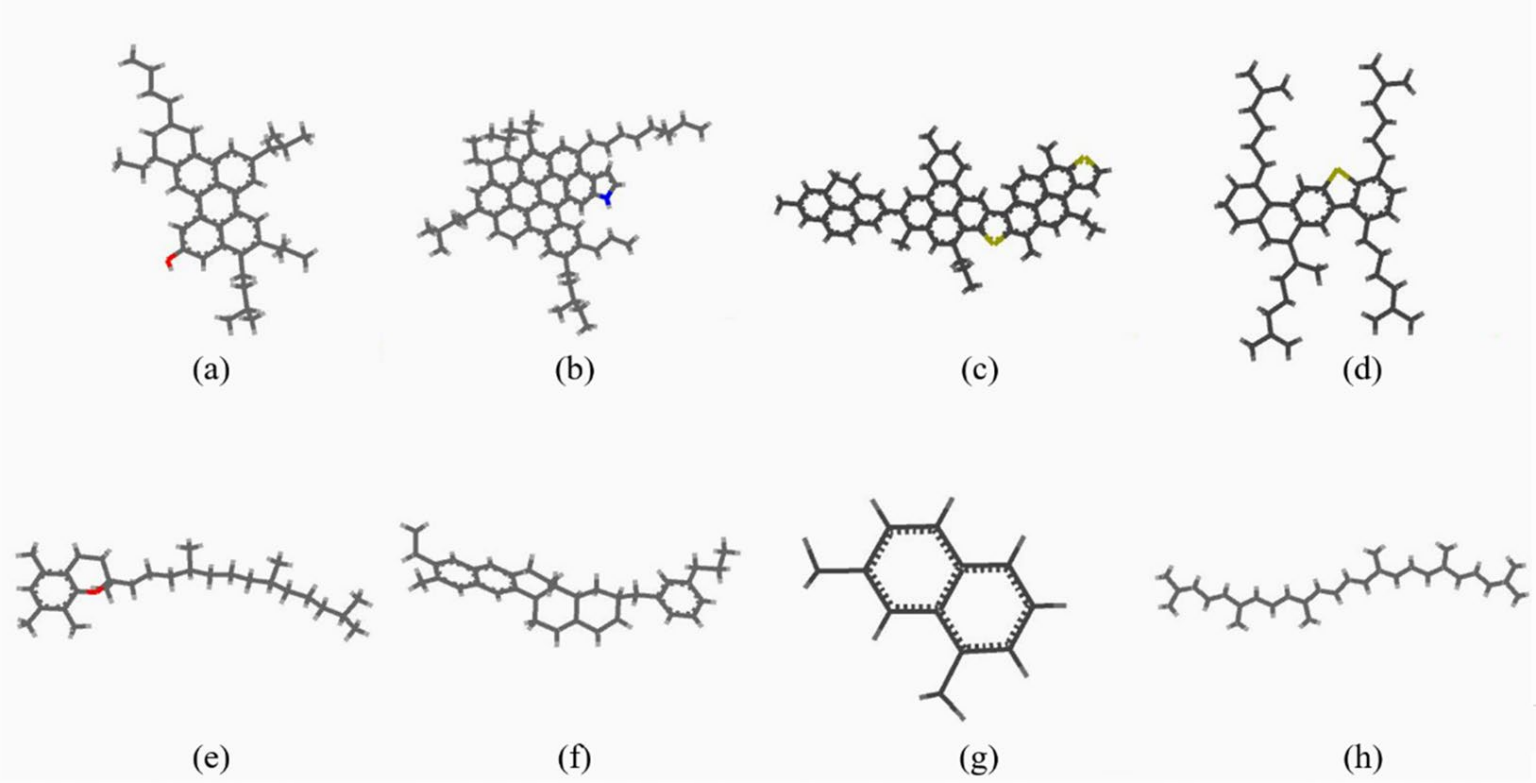
Selected molecular models of asphalt components. (**a**) Asphaltene 1^23^, (**b**) Asphaltene 2^23^, (**c**) Asphaltene 3^11^, (**d**) Resin 1^32^, (**e**) Resin 2^23^, (**f**) Aromatic 1^35^, (**g**) Aromatic 2^7^, (**h**) Saturate^36^ (carbon atom is dark grey, oxygen atom is red, sulfur atom is yellow and nitrogen atom is blue).

Asphaltene is the most complicated and heaviest component in asphalt binder. Based on the solubility concept, asphaltenes are generally defined as the fraction in petroleum, which cannot be dissolved in normal saturated alkanes with low molecular weight (such as n-heptane and n-pentane) but can be soluble in aromatic hydrocarbons (such as toluene)^31^. The choice of the asphaltene model for this study was derived from the research of Zhang^7^ and Li^23^. Asphaltene 1 proposed by Artok et al. and asphatene 2 proposed by Groenzin and Mullins were selected in Zhang’s research. Asphaltene1 consists of an aromatic core and a shorter side chain while the side chain of asphaltene 2 is longer than aphaltene1. Mullins model is an improvement of Yen model and reflects the structure of asphaltene molecules better than the model in the earlier study of Groenzin and Mullins^13,14^. Three different asphaltene models in the Mullins study were chosen and slightly modified the locations of the side group by Dreck. The original Mullis model has higher internal energy due to the “pentane effect”, thus it can be effectively alleviated the internal energy by adjusting the position of the side group appropriately^23^. According to the asphaltene model proposed by Dreck and Zhang, three model molecules were selected in this study as the asphaltene fraction. All selected model of the asphalt components is shown in Fig. 1.

### Resin

Asphaltenes are coated by resins and connected by maltenes to form the structure of the asphalt binder. Besides, the source of asphalt binder ductility is that the rising temperature makes the original solid or semi-solid resin fraction liquid, and the flow of resin disperses the viscous asphaltenes. There are more polar molecular in resin than those in asphaltene, thus the most polar component in asphalt binder is resin. Two molecular models from the literature were selected in this study to represent resin fraction in the asphalt model. Resin 1 and Resin 2 are shown in Fig. 1. The resin 1 with heteroatom sulfur, was detected from the Venezuela crude oil successfully applied in related studies of Molecular Dynamics (MD) simulation^32,33^. Resin 2 has been shown in Dreck’s research to effectively represent the resin components in the asphalt molecular model.

### Aromatics

This component is the less polar part of asphalt, mainly composed of alkanes, naphthene, and aromatic compounds. The choice of aromatics molecular model for this study is derived from research by Dreck and Zhang. Two molecular of naphthene aromatics named perhydrophe-nanthrene-naphthalen (PHPN) and dioctyl-cyclohexane-naphthalene (DOCHN) were proposed by Lira-Galeana et al. and Simanzhenkov et al. in an early study^34,35^. And these molecular models have been utilized through the MD simulation method by Dreck. PHPN contains both aromatic rings and naphthenic rings and was selected in this study as an aromatic fraction. Zhang showed 1,7-dimethylnaphthalene as the aromatic component, which was chosen as another aromatic model (Fig. 1) in this study.

### Saturates

Saturation fraction is a relatively simple component in asphalt, which has no polarity. The saturates is the most valuable fraction of the oil industry, and it is mostly composed of normal paraffins, iso-paraffins, and naphthene. The molecular selected to represent the saturate fraction in this study is squalene (Fig. 1), which has been identified in petroleum and can be extracted from animals and plants^36^.

As is well known that the composition of asphalt binder is extremely complex, and the types and quantities of compounds with different components are also very large. The content of each fraction and composition ratio of compounds are different from different habitats. Therefore, this study proposed an average molecular model based on literature data, rather than a specific asphalt binder. In SARA four fractions of asphalt, the experimental proportion range of each component is as follows: 1.9%-19.1% saturate, 22.4%-46.6% aromatic, 18.7%-52.7% resin, and 7.8%-25.6% asphaltene^37^. On this basis, the average percentage of each component is considered, and finally, the asphalt model used in this study is proposed.

### Molecular dynamics simulation

Molecular structure and microscopic interaction are regarded as two entry points for computational simulation to study material properties. This method was provided by Alder and Wainwright, and subsequently perfected in the work of Verlet, Anderson, and Nose et al., and finally realized the thermostatic molecular dynamics method^38–41^. The procedure of MD simulation is usually divided into three steps. The structure and potential of the model system should be considered in the first step. Then, the movement of individual particles can be calculated based on Newton’s equation until the properties of the model system are stable. Finally, the simulation data was analyzed to predict the physical properties of the model. NPT ensemble and NVT ensemble were adapted in different simulation procedures of this study, respectively. In addition, another MD simulation method named geometry optimization (also named energy minimization) has been used in this research to optimize the initial geometry structure and minimize the primary energy of the molecular system.

The DREIDING Force Field and AMBER Cornell Extension Force Field were implemented to predict system density and verify the asphalt model in this study. And the AMBER Cornell Extension Force Field was applied to calculate further properties of the asphalt molecular model. DREIDING Force Field is a simple but universal force field, that has been proved to be useful for predicting the structure and dynamics of organic, biological, and main-group inorganic molecules. In DREIDING Force Field, potential energy is defined as a superposition of valence intersection(bond) and non-bond intersection [Eq. (1)]. And the definition of valence intersection and the non-bond intersection is shown in Eq. (2) and Eq. (3), respectively^39^.

1 $$E={E}_{val}+{E}_{nb}$$

where *E* is potential energy, *E*_*val*_ is valence intersection and *E*_*nb*_ is the non-bond intersection.

2 $${E}_{val}={E}_{B}+{E}_{A}+{E}_{T}+{E}_{I}$$

where *E*_*B*_ is bond stretch for two-body, *E*_*A*_ is bond-angle bend for two-body, *E*_*T*_ is dihedral angel torsion and *E*_*I*_ is inversion terms for four body.

3 $${E}_{nb}={E}_{vdw}+{E}_{Q}+{E}_{hb}$$

where *E*_*vdw*_ is van der Waals intersection and dispersion, *E*_*Q*_ is electrostatic and *E*_*hb*_ is explicit hydrogen bonds.

To simulate proteins, nucleic acid, and organic molecules, the AMBER (Assisted Model Building with Energy Refinement) Force Field with fewer molecular parameters was first developed. Besides, a general AMBER Force Field (GAFF) was proposed that applies to more molecules and may be compatible with traditional AMBER Force Field^42^. The structures and bond/non-bond intersection in AMBER Cornell Force Field and GAFF are described by Eq. (4). The parameters of GAFF were added to AMBER Cornell Force Field, the new force field was applied in this study called AMBER Cornell Extension Force Field^43,44^.

4 $$ E_{total} = \mathop \sum \limits_{bonds} K_{r} \left( {r - r_{eq} } \right)^{2} + \mathop \sum \limits_{angles} K_{\theta } \left( {\theta - \theta_{eq} } \right)^{2} + \mathop \sum \limits_{dihedrals} \frac{{V_{n} }}{2}\left[ {1 + \cos \left( {n\varphi - \gamma } \right)} \right] + \mathop \sum \limits_{i < j} \left[ {\frac{{A_{ij} }}{{R_{ij}^{12} }} - \frac{{B_{ij} }}{{R_{ij}^{6} }} + \frac{{q_{i} q_{j} }}{{ \in R_{ij} }}} \right] $$

where the term 1–4 means the energy of bond, angle, dihedral, and non-bond intersection. *r*_*eq*_ and *h*_*eq*_ are the equilibrium structural parameters; *K*_*r*_ and *K*_*h*_ are the force constants; n and *γ* are the multiplicity and phase angle for the torsional angle parameters, respectively; A, B, and q are the non-bonded potentials between all-atom pairs; and finally, *R*_*ij*_ and ϵ are the distances of the atoms and well depth for the van der Waals energy calculation.

In addition, the Ewald summation was chosen to calculate the electrostatic forces of molecular systems in this study. The basic idea of Ewald summation is to represent the electrostatic in periodic space by the summation of the charge interactions in the periodical simulation cell. And this method converts a sum that converges slowly or conditionally through a Fourier transform and a convergent function to two sums that converges quickly. So, the electrostatic potential is divided into long-range potential which is calculated in real space, and short-range potential which is calculated in k space by Fourier transform^33,45^.

### Optimization method

The energy of built molecular models is necessary to optimize due to the high potential energy that would adversely affect the simulation calculation. Firstly, the energy of each established model is required to minimize. Secondly, the amorphous cell built by placing the molecular model of different components in the periodic boundary will also need to optimize. Compared to the existing optimization methods, the conjugate gradient method and Broyden–Fletcher–Goldfarb–Shanno (BFGS) method (a kind of quasi-Newton method) have been selected in this study. The different forms of conjugate gradient method can be used for the solution of small and simple systems or the simulation and optimization of large sparse systems^3^. The computing equations of the direct and iterative method of the conjugate gradient method are shown as Eqs. (5) and Eq. (6) ^46^.

5 $$Ax=b, A\in {R}^{n\times n}\,and\,{u}^{T}Av=0, (direct \,  method)$$

6 $$f\left(x\right)=\frac{1}{2}{x}^{T}Ax-{x}^{T}b, x\in {R}^{n}, (iterative \,  method)$$

where *A* is real symmetric matric; *b* is a coefficient, and vectors *u* and *v* are non-zero.

Both the conjugate gradient method and the quasi-Newton method require the calculation of the gradient of the function or the first derivative of any point. However, the data storage, information update, and memory requirement of the two methods are different. The calculation formulae of the BFGS method [Eqs. (7)–(11)]^46,47^ are shown below.

7 $$g\left(x\right)=\nabla f\left(x\right)=0$$

The minimum is obtained for Eq. (7). Where the *f(x)* is the function to be minimization and *x* = (*x*_1_,…, *x*_*nc*_)^*T*^.

Then, the Newton method achieves quadratic convergence through iterative calculation.

8 $${x}_{k+1}={x}_{k}-{H}_{k}^{-1}{g}_{k}$$

9 $${g}_{k}=\nabla f\left({x}_{k}\right); {H}_{k}=H\left({x}_{k}\right)={[\frac{{\partial }^{2}f}{\partial {x}_{i}\partial {x}_{j}}]}_{n\times n}$$

where *x*_*k*_ means the vector x at the kth iteration; *g*_*k*_ is the gradient vector and *H*_*k*_ is the Hessian matrix of the function *f*(*x*).

The BFGS method approximates the Hessian matrix with matrix *B* based on function *f* and gradient *g*, and the calculation formula is shown as Eqs. (10) and (11).

10 $${B}_{k+1}={B}_{k}+\frac{{y}_{k}{y}_{k}^{T}}{{y}_{k}^{T}{p}_{k}}-\frac{{{B}_{k}{p}_{k}({B}_{k}{p}_{k})}^{T}}{{p}_{k}^{T}{B}_{k}{p}_{k}}$$

$${y}_{k}=\nabla f\left({x}_{k}\right)-\nabla f\left({x}_{k-1}\right); {p}_{k}={x}_{k}-{x}_{k-1}$$

11 $${B}_{k+1}^{-1}=\left(1-\frac{{p}_{k}{y}_{k}^{T}}{{y}_{k}^{T}{p}_{k}}\right){B}_{k}^{-1}\left(1-\frac{{y}_{k}{p}_{k}^{T}}{{y}_{k}^{T}{p}_{k}}\right)+\frac{{p}_{k}{p}_{k}^{T}}{{y}_{k}^{T}{p}_{k}}$$

The BFGS method and conjugate gradient method were used to optimize the system energy of the asphalt model and compare that which one is more suitable for the proposed asphalt molecular model.

**Figure 2:**
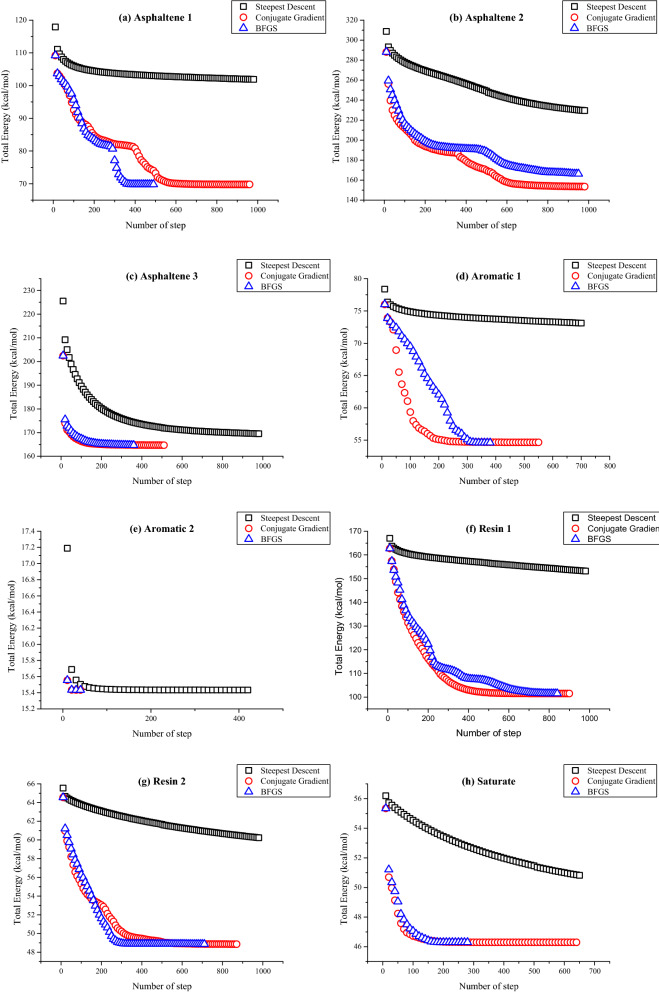
Energy optimization of asphalt model [(**a**–**h**) indicates the total energy changes of different molecules under the three optimization methods].

**Figure 3:**
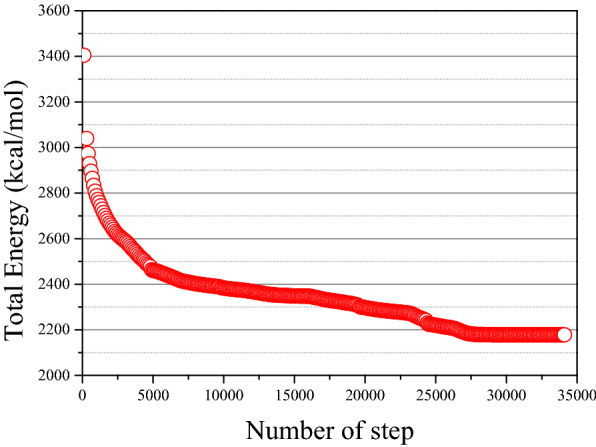
Energy optimization of the asphalt model.

The results of energy optimization for different components of molecules in three optimized methods are shown in Fig. 2. The energy minimization effect of the steepest descent method with 500 steps is worse than that of the other two methods. As shown in Fig. 2, after 500 steps of optimization by the steepest descent method, the total energy reduction of single molecules is limited and does not reach a stable state. However, the conjugate gradient method and the BFGS method have a better result of optimization, and successfully minimize the energy of single molecules. Besides, in the optimization procedure of the same molecular model, the number of steps, convergence speed, and minimum values required by these two methods are different. For instance, after 350 steps of BFGS optimization, the energy of asphaltene 1 is stable at the calculated minimum value: 69.8 kcal/mol, while the total energy is still high and unstable after 500 steps optimization by conjugate gradient method. Similar differences are observed in asphaltene 2, aromatic 1, resin1, and resin 2. Therefore, these two methods were applied in this study to optimized different components and molecules in order to adjust the initial conformation of the asphalt model. BFGS method was used to optimize asphaltene 1 and resin 2, and other molecules were optimized by the conjugate gradient method. In addition, the amorphous structure representing the molecular model of asphalt binder has been optimized by these two methods. As shown in Fig. 2, after enough optimization steps in both methods, only asphaltene1 was found to have a lower energy value of BFGS than the conjugate gradient method, while the value of other molecules optimized by the conjugate gradient method is lower or equal to that of the BFGS method. So, the optimized method for the asphalt model in this study is that the conjugate gradient method is used for 30,000 steps first, and then the BFGS method is applied for the last 5000 steps. It can be shown in Fig. 3 that the total energy of the optimized asphalt model finally stabilizes around 2178 kcal/mol.

### Simulation detail

The molecular models used in this study have been shown in the first section of this chapter. The initial number of each molecular in this system is shown in Table 1. The periodical boundary condition (PBC) is applied in three directions. Under the periodic boundary condition, the corresponding surfaces (such as the right surface and left surface) in the simulation area will be connected to each other to form a path. Particles passing through the unit cell on one side will reappear in the same form on the other side. The initial density of the asphalt model is 0.1 g/cm^3^ because the distribution of components can be more random at low density. It is also convenient for subsequent position adjustment and energy optimization.

**Table 1:** Composition and proportion of asphalt model.

Molecular model	Sum formula	Number of molecules	Molecular weight	Proportion
Asphaltene 1	C_42_H_54_O	3	1722	0.23
Asphaltene 2	C_66_H_81_N	2	1774	
Asphaltene 3	C_64_H_52_S_2_	2	1768	
Resin 1	C_49_H_78_S	5	3490	0.22
Resin 2	C_29_H_50_O	4	1656	
Saturate	C_30_H_62_	15	6330	0.28
Aromatic 1	C_35_H_44_	10	4640	0.27
Aromatic 2	C_12_H_12_	10	1560	
Asphalt model	C_1667_H_2508_N_2_O_7_S_9_	1	22,940	

Based on the obtained molecular model and proportion, the asphalt model was built using the DREIING Force Field and AMBER Cornell Extension Force Field. Then, all molecules were placed in an amorphous model with a periodical boundary condition. Once the model system was developed with the selected force field, the conjugate gradient method was applied to minimize the initial energy and optimize the geometry structure. Finally, several MD simulation procedures were carried out under the NPT ensemble to predict the density of the model system and calculate properties.

**Figure 4:**
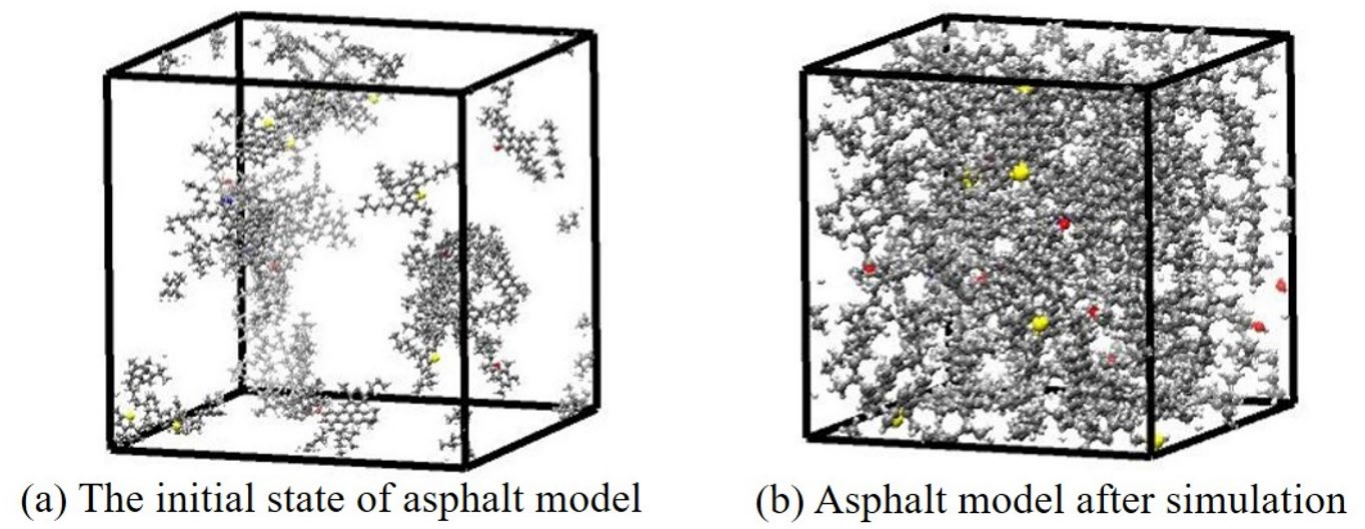
Molecular image of asphalt model at different state.

**Figure 5:**
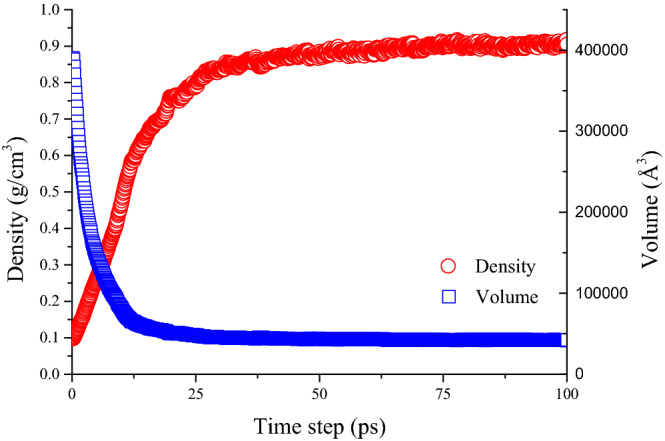
Relationship of simulation cell volume and system density.

The asphalt model was equilibrated in three steps. Firstly, using the conjugate gradient method to optimize the energy of the model system at 5000 max iteration. Secondly, run 200 ps at 100 atm and 298.15 K. Thirdly, subject the model system to 1 atm and 298.15 K for 1000 ps. The minimum time step of the simulation procedure is 1 fs, and the trajectories were recorded every 100 fs. Besides, the Nose–Hoover thermostat and barostat were used during the NPT simulation to maintain the temperature and pressure of the model system at 298.15 ± 10 K and 1 atm, respectively. Under the high-pressure condition, the molecules in the system accelerate position adjustment and quickly find more suitable geometric positions. The purpose of the third step is to make the asphalt model reach a stable state by carrying out sufficient time at room temperature and normal pressure. After these steps, the model system reaches relative equilibrium, and the state of the asphalt model is shown in Fig. 4. Figure 4 shows the different states of the asphalt model before and after the NPT simulation. The asphalt molecules distribute loosely in the unit cell at a lower density before the simulation, while the cell is more tightly packed after the simulation. As is shown in Fig. 5, the reason for this phenomenon is that the initial density of the asphalt model is lower than the real value in order to disperse the asphalt component molecules in the simulation cell. In the subsequent simulation process, the volume of the simulation cell gradually decreases, while the model density approaches the real value.

## Result and analyze

### Density prediction

**Figure 6:**
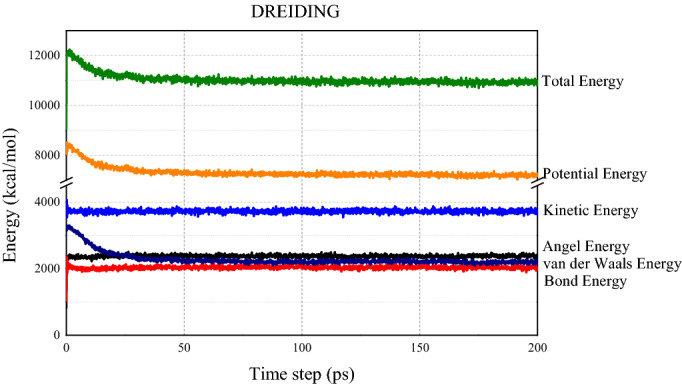
System energy after 200 ps NPT simulation under DREIDING force field.

**Figure 7:**
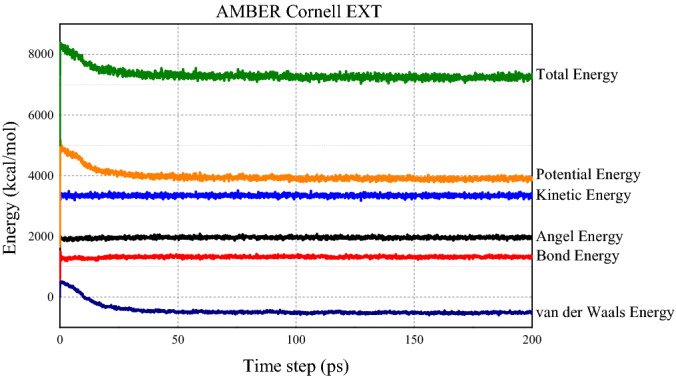
System energy after 200 ps NPT simulation under AMBER Cornell extension force field.

The stable state of the model system depends on the energy of the system. As shown in Figs. 6 and 7, at the beginning of the simulation, the total energy, potential energy and van der Waals energy of the molecular systems maintain at a high value. During the first 20 ps time steps, energies in the system drop dramatically. As the simulation progresses, the total energy, potential energy, and van der Waals energy gradually decrease and eventually stabilize at 7200 ~ 7400 kcal/mol, 3700 ~ 3900 kcal/mol, and − 450 ~ − 550 kcal/mol (AMBER Cornell Extension Force Field), 10,800 ~ 11,000 kcal/mol and 7000 ~ 7200 kcal/mol and 2100 ~ 230 kcal/mol (DREIDING Force Field), respectively.

It is necessary to calculate the density of the model system to verify the validity and representativeness of the asphalt model. Density is a commonly used method to verify the model system in MD simulations, and it is also effective to match the real asphalt binder. The density of asphalt is closely related to its chemical composition and internal structure, which depends on the proportion and arrangement of the components inside the asphalt. And density is also the evaluation index of asphalt binder quality performance. Therefore, in this research, the density of the asphalt model is predicted by MD simulation in different force fields. The predictive value at room temperature (298.15 K) and normal pressure (1 atm) is compared with the actual asphalt to evaluate the effectiveness of the model. The model which is closer to the actual value is selected for subsequent performance calculation.

**Figure 8:**
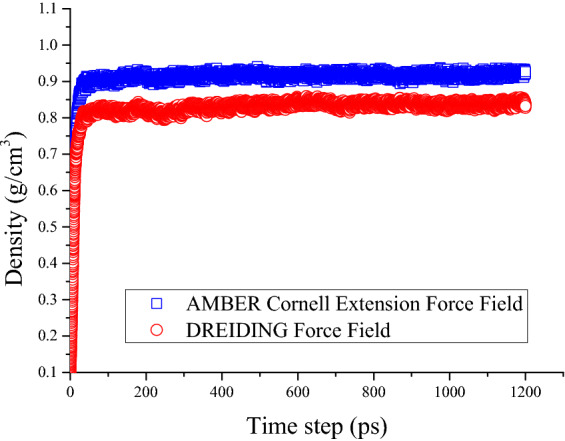
Density of the asphalt model.

Figure 8 shows the density results after three simulation steps in AMBER Cornell Extension Force Field and DREIDING Force Field. As shown in Fig. 8, the density value of the model system increased sharply in the first 100 ps and gradually stabilizes in the subsequent simulation process. The mean value within this range is the density prediction of the model asphalt. Asphalt models in both force fields have similar trends over the simulation time, but the final stable values are different. This is caused by different parameter settings for the functional groups in the force field. The prediction density value of DREDING Force Field and AMBER Cornell Extension Force Field is around 0.84 and 0.92 g/cm^3^, respectively. However, the experimental value of the density of the asphalt binder ranges from 0.95 to 1.15 g/cm^3^. The density value of model asphalt is smaller than the real value of asphalt binder, especially in DREIDING Force Field. The proportion of components was adjusted to make the density of model asphalt more consistent with the actual value. The DREIDING Force Field will not be considered in subsequent calculation due to the unacceptable density error, and the AMBER Cornell Extension Force Field is applied to verify the adjustment model.

### Model adjustment

**Figure 9:**
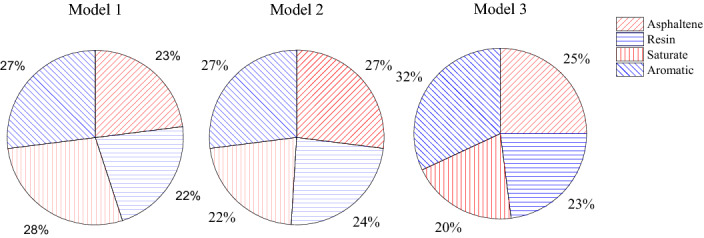
The percentage of four components in three models.

The proportions of components in model asphalt molecules were adjusted and the density values were predicted. The differences between the two adjusted asphalt models and the original molecular model are shown in Table 2. And the change in the content of four components in the three asphalt models is shown in Fig. 9.

**Table 2:** Comparison of the model parameter before and after adjustment.

Molecular model	Model 1	Model 2	Model 3
Asphaltene 1	3	3	3
Asphaltene 2	2	2	2
Asphaltene 3	2	2	3
Resin 1	5	4	5
Resin 2	4	5	5
Saturate	15	10	12
Aromatic 1	10	11	10
Aromatic 2	10	10	10
Formula	C_1667_H_2508_N_2_O_7_S_9_	C_1532_H_2214_N_2_O_8_S_8_	C_1670_H_2424_N_2_O_8_S_11_

**Figure 10:**
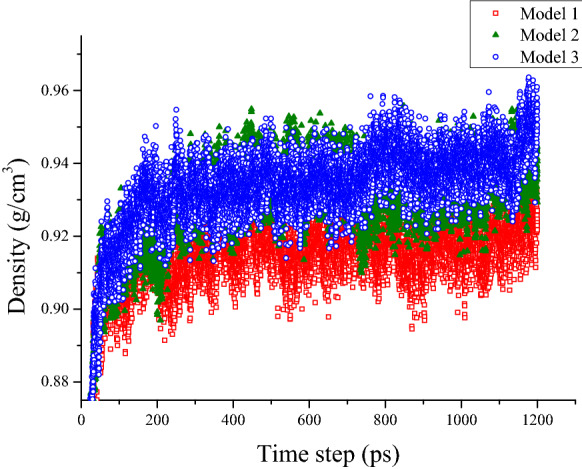
Density of original model and adjusted models.

**Figure 11:**
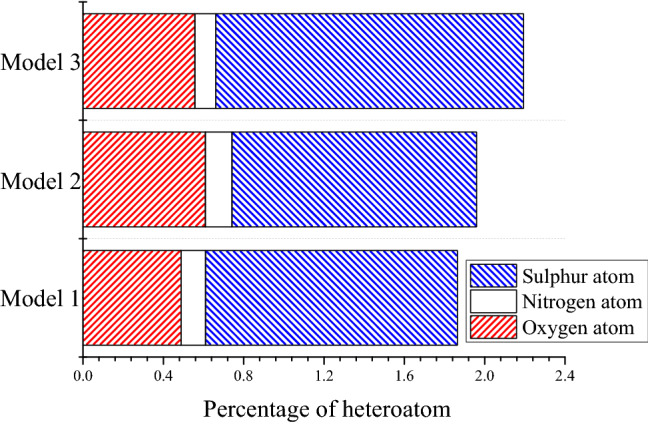
The percentage of each heteroatom in three models.

**Figure 12:**
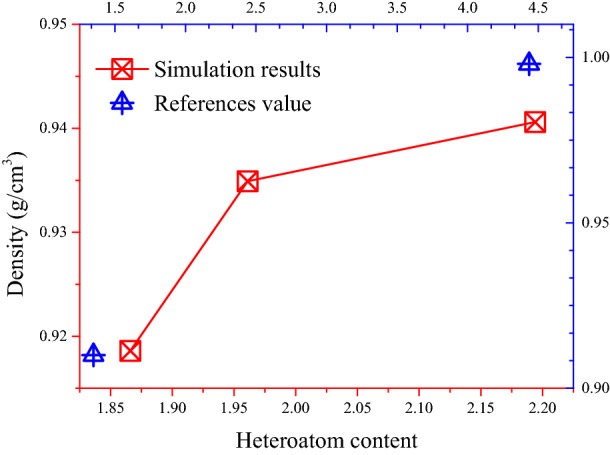
The relationship between Heteroatom content and system density.

The density of these three models is shown in Fig. 10. Obviously, the density of asphalt changed and was closer to the experimental value by adjusting the number of different molecules and the proportion of fraction of the asphalt model. The density average value of Model 3 and Model 2 approaches 0.95 g/cm^3^, which is higher than the original Model 1. The carbon-hydrogen ratio and mass fraction of each element are listed in Table 2, and the influence factors on asphalt density were explored by analyzing the variation of these values in three models. Among all the indexes, the carbon-hydrogen, the mass percentage of oxygen atoms, and the content of heteroatoms in Model 2 and Model 3 are all higher than Model 1. There were also small differences in other parameters, but no significant difference between the models before and after adjustment. As shown in Fig. 11, the total content of heteroatom gradually increased in Model 1, Model 2, and Model 3, although this value of each atom (oxygen, Nitrogen, and Sulphur) was not always increased during the adjustment. After running the 500 ps NPT simulation, the mean densities for the last 200 ps were selected to discuss the relationship between heteroatom content and system density. It can be shown in Fig. 12 that the density value of the asphalt model was increased with the increase of heteroatom content. When the total heteroatom proportion increased to 2.2%, the average density value of the model finally reached 0.94 g/cm^3^. Besides, the relationship between heteroatom content and density in reference has been calculated and shown in Fig. 12. The density of the asphalt model is affected by the number of heteroatoms. Although there may be more complicated reasons for this effect, this result makes it worthy of attention.

### MD prediction of the glass transition temperature

Asphalt binder is a temperature-sensitive material, and the properties will change with the temperature variation. In three temperature stages: low-temperature, medium-temperature, and high-temperature, the behaviors of the asphalt binder were divided into the Newtonian flow, viscoelastic state, and vitrification state respectively. It is an important property of the asphalt binder. And the definition of the glass transition is the process of asphalt from a viscoelastic state to a vitrification state. The intermolecular motion of material at the micro-level and the basic mechanical properties at the macro level changed greatly through the glass transition, but it is different from phase change. Glass transition temperature has a great influence on the properties of asphalt binder, especially on the low-temperature behavior, due to the significant physical hardening of the asphalt binder was found near the glass transition temperature. And the physical hardening can lead to brittle cracking of asphalt binder^48^.

**Figure 13:**
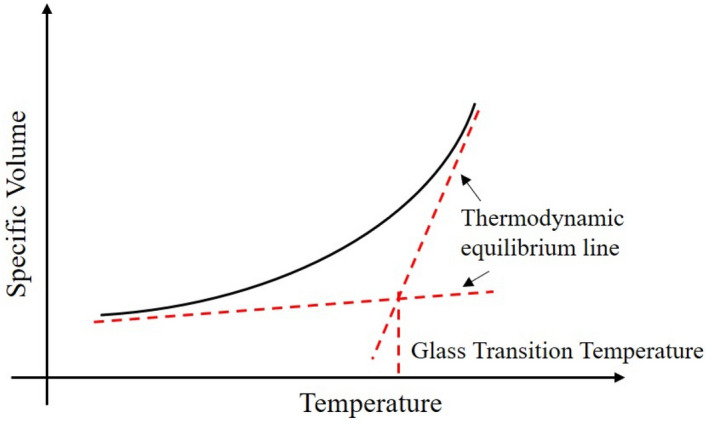
The temperature region of the glass transition.

**Figure 14:**
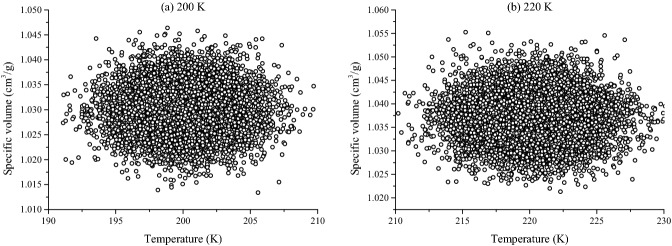
The specific volume of the asphalt model 3 at different temperature (10,000 points).

The specific volume of a material is defined as the ratio of volume to mass, which is numerically equal to the inverse of the density. The specific volume-temperature curve is shown in Fig. 13, and the two asymptotic lines of the curve are also called thermodynamic equilibrium lines. The intersection of these two lines is defined as the glass transition temperature. Multiple glass transitions can be observed in the asphalt binder when the temperature rises or falls. This phenomenon can be attributed to the complex composition of the asphalt binder. And the glass transition temperature of asphalt is the result of several transitions of different components. According to the data of reference, the glass transition behavior of asphalt binder may occur within a wide temperature range: − 50 °C–30 °C (223.15 K–303.15 K)^49^. In this study, the temperature range of MD simulation was set at 200.15 K–400.15 K, and the specific volume of asphalt model 3 at 200.15 K and 220.15 K is shown in Fig. 14. The specific volume fluctuates over a given temperature range and can be calculated by averaging these data to obtain the specific volume at the given temperature, such as results at 200.15 K in the figures. The figures at other temperatures are not presented in this paper because of space constraints.

**Figure 15:**
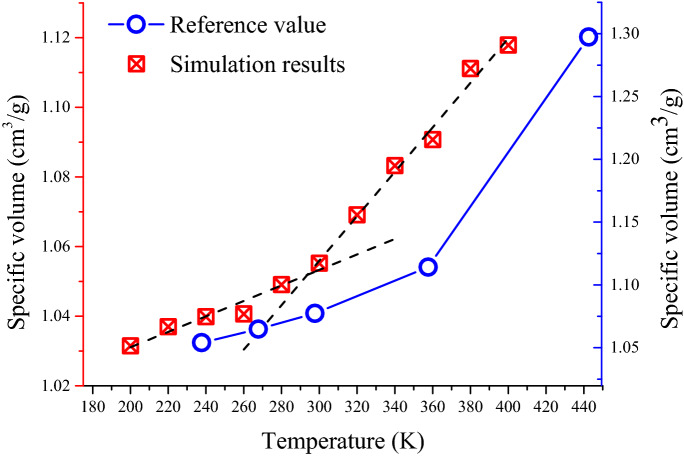
The relationship between the specific volume and temperature at different temperatures in the asphalt model 3. (The left is the ordinate of experimental/simulation value, and the right is the ordinate of the reference value).

The specific volume averages at different temperatures were calculated and plotted a curve as shown in Fig. 15. The relationship between the specific volume and temperature of asphalt model 3 can be clearly observed from Fig. 15. With the gradual increase of temperature, the specific volume also increases. Base on the definition of glass transition, an interval where the slope changed significantly should be found in the curve. Ten temperatures have been chosen in MD simulation to calculate the relationship of specific volume and temperature. And the selected temperature value is 220.15 K, 240.15 K, 260.15 K, 280.15 K, 300.15 K, 320.15 K, 340.15 K, 360.15 K and 380.15 K. During the MD simulation, the temperature raised 20 K per procedure, and run 200 ps under the NPT ensemble to predict the glass transition temperature of the asphalt model. As is shown in Fig. 15, the linear regression analysis shows that the change of specific volume with temperature in the range of 200 K–280 K and 320 K–380 K can be regarded as a directly proportional relationship. The slope of the temperature-specific volume curve changed significantly within the temperature range of 280 K–320 K, which can be identified as the temperature interval of the glass transition. In addition, it can be predicted by the intersection point of the two fitting lines that the glass transition temperature of the asphalt binder is around 300 K. This result is consistent with the experimental value, and the more accurate range and predictive value are given in this study.

## Conclusion

An asphalt model that contains eight molecules selected from literature, was proposed in this experimental MD simulation. The AMBER Cornell Extension Force Field was applied to build models and calculate the properties. The conjugate gradient method and quasi-newton method were used to optimize the geometry of the asphalt model. The properties of the asphalt model were calculated including density and glass transition temperature. The computation result and discussion can be drawn:

1. Eight molecules were selected to represent four fractions of asphalt binder including saturates, aromatics, resins, and asphaltenes. The BFGS (a kind of quasi-newton method) was applied to optimize asphaltene 1 and resin 2. Other molecules were optimized by the conjugate gradient method. Then, the asphalt model with a periodical boundary cell was optimized 30,000 steps by conjugate gradient method and 5000 steps by BFGS method.
2. The density of the original asphalt model was calculated in 298.15 K using DREIDING Force Field and AMBER Cornell Extension Force Field to select a more suitable force field for properties calculation and analysis. The AMBER Cornell Extension Force Field was chosen to calculate the density value of the original model and adjusted model. The simulation results show that the density of the asphalt model was affected by the carbon-hydrogen ratio, percentage of heteroatoms, and asphaltene proportion. In the three models, the heteroatom content increased from 1.866 to 2.194, and the system density of these models also increased gradually in the process. Besides, the density of the asphalt molecular system also can be impacted by the increased mass fraction of polar components such as resins and asphaltene.
3. Based on the relationship between specific volume and temperature, the glass transition temperature was calculated. The glass transition behavior of asphalt binder can be observed in the temperature of 280 K–320 K according to the experimental data. The prediction value of glass transition temperature in this study is 300 K.
